# Bis(μ-4-chloro-2-oxidobenzoato)bis­[(1,10-phenanthroline)copper(II)] dihydrate

**DOI:** 10.1107/S1600536810008354

**Published:** 2010-03-10

**Authors:** Jing-Jing Nie, Jun-Hua Li, Duan-Jun Xu

**Affiliations:** a Department of Chemistry, Zhejiang University, People’s Republic of China

## Abstract

The structure of the the title compound, [Cu_2_(C_7_H_3_ClO_3_)_2_(C_12_H_8_N_2_)_2_]·2H_2_O, consists of a dimeric unit involving a planar Cu_2_O_2_ group arranged around an inversion center. The coordination sphere of the Cu^II^ atom can be described as an elongated distorted square pyramid where the basal plane is formed by the two N atoms of the 1,10-phenanthroline mol­ecule and the two O atoms of the hydroxy­chloro­benzoate (hcbe) anion. The long apical Cu—O distance of 2.569 (2) Å involves the O atom of a symmetry-related hcbe anion, building up the dinuclear unit. Each dinuclear unit is connected through O—H⋯O hydrogen bonds involving two water mol­ecules, resulting in an *R*_4_^2^(8) graph-set motif and building up an infinite chain parallel to (10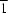). C—H⋯O inter­actions further stabilize the chain.

## Related literature

For our ongoing investigation of the nature of π–π stacking, see: Su & Xu (2004 ▶); Xu *et al.* (2007 ▶). For related structures, see: Yang *et al.* (2006 ▶); Garland *et al.* (1987 ▶); Li *et al.* (1995 ▶); Fan & Zhu (2005 ▶); Song *et al.* (2007 ▶). For a structural discussion on hydrogen bonding, see: Etter *et al.* (1990 ▶); Bernstein *et al.* (1995 ▶).
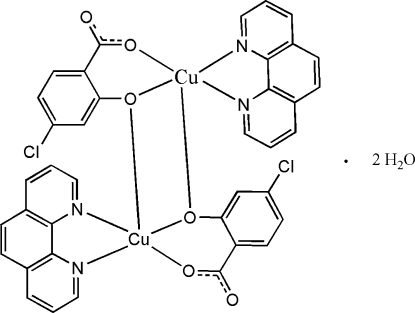


## Experimental

### 

#### Crystal data

[Cu_2_(C_7_H_3_ClO_3_)_2_(C_12_H_8_N_2_)_2_]·2H_2_O
*M* *_r_* = 864.60
Monoclinic, 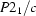

*a* = 8.1941 (17) Å
*b* = 18.851 (4) Å
*c* = 11.873 (3) Å
β = 105.993 (8)°
*V* = 1763.0 (6) Å^3^
*Z* = 2
Mo *K*α radiation
μ = 1.42 mm^−1^
*T* = 294 K
0.33 × 0.30 × 0.22 mm

#### Data collection

Rigaku R-AXIS RAPID IP diffractometer
Absorption correction: multi-scan (*ABSCOR*; Higashi, 1995 ▶) *T*_min_ = 0.656, *T*_max_ = 0.730
18894 measured reflections
3163 independent reflections
2162 reflections with *I* > 2σ(*I*)
*R*_int_ = 0.047

#### Refinement

*R*[*F* ^2^ > 2σ(*F* ^2^)] = 0.037
*wR*(*F* ^2^) = 0.103
*S* = 1.03
3163 reflections
244 parameters
H-atom parameters constrained
Δρ_max_ = 0.58 e Å^−3^
Δρ_min_ = −0.36 e Å^−3^

### 

Data collection: *PROCESS-AUTO* (Rigaku, 1998 ▶); cell refinement: *PROCESS-AUTO*; data reduction: *CrystalStructure* (Rigaku/MSC, 2002 ▶); program(s) used to solve structure: *SIR92* (Altomare *et al.*, 1993 ▶); program(s) used to refine structure: *SHELXL97* (Sheldrick, 2008 ▶); molecular graphics: *ORTEP-3 for Windows* (Farrugia, 1997 ▶) and *PLATON* (Spek, 2009 ▶); software used to prepare material for publication: *WinGX* (Farrugia, 1999 ▶).

## Supplementary Material

Crystal structure: contains datablocks I, global. DOI: 10.1107/S1600536810008354/dn2542sup1.cif

Structure factors: contains datablocks I. DOI: 10.1107/S1600536810008354/dn2542Isup2.hkl

Additional supplementary materials:  crystallographic information; 3D view; checkCIF report

## Figures and Tables

**Table 1:** Hydrogen-bond geometry (Å, °)

*D*—H⋯*A*	*D*—H	H⋯*A*	*D*⋯*A*	*D*—H⋯*A*
O1*W*—H1*A*⋯O2	0.90	1.92	2.817 (4)	175
O1*W*—H1*B*⋯O2^i^	0.88	2.13	2.921 (4)	150
C10—H10⋯O2^ii^	0.93	2.42	3.277 (5)	153
C17—H17⋯O1*W*^i^	0.93	2.58	3.487 (4)	166

Symmetry codes: (i) 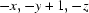; (ii) 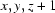.

**Fig. 1.**
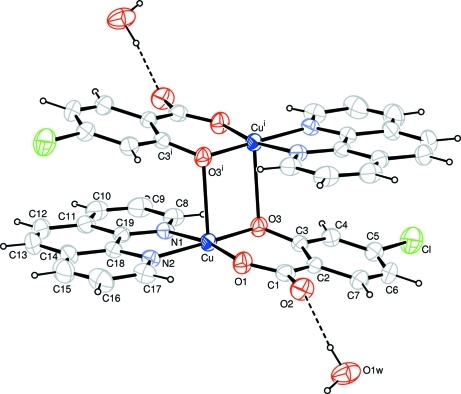
The dinuclear molecular structure of the title compound with 30% probability displacement (arbitrary spheres for H atoms) [symmetry code: (i) 1-x, 1-y, 1-z].

**Fig. 2.**
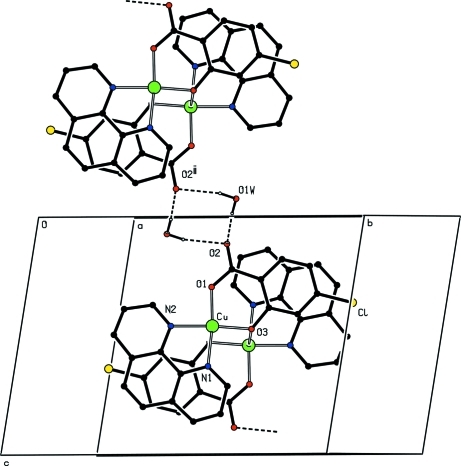
Partial packing view showing the formation of the chain through the O-H···O hydrogen bonds. H atoms not involved in hydrogen bondings have been omitted for clarity. H bonds are shown as dashed line.[Symmetry codes: (ii) -x, -y+1, -z]

## supplementary crystallographic information

### Comment

As part of our ongoing investigation on the nature of π-π stacking (Su & Xu, 2004; Xu *et al.*, 2007), the title Cu^II^ compound incorporating 2-hydroxy-4-chlorobenzoate (hcbe) ligand has recently been prepared in the laboratory and its crystal structure is reported here.

The structure of the the title compound, (C_19_H_11_ClCuN_2_O_3_).(H_2_O), consists of a dimeric unit involving a planar Cu_2_O_2_ group arranged around inversion center The coordination sphere of the Cu^II^ can be described as an elongated distorted square pyramid where the basal plane is formed by the two N atoms of the 1,10-phenanthroline molecule and the two O atoms of the hydroxychlorobenzoate (hcbe) anion. The long apical Cu-O3 distance of 2.569 (2)A involves the O3 atom of the symmetry related hcbe anion [symmetry code (i) i-x,1-y,1-z] building up the dinuclear unit (Fig. 1).

This apical Cu-O3 distance is 0.674 (3) Å longer than Cu—O3 bond distance in the basal coordination plane, showing the Jahn-Teller distorted square-pyramidal coordination geometry around the Cu^II^ cation. A patially overlapped arrangement is observed between the nearly parallel C2-C7 phenyl ring and C11-C19 phen ring system [dihedral angle 14.25°]. The centroid to centroid distance is 3.649 (3) Å and the perpendicular distance of the centroid to the rings is 3.456 and 3.571 Å respectively suggesting a weak π-π stacking comparable to that found in the related Cu^II^ complexes (Garland *et al.*, 1987; Li *et al.*, 1995; Fan & Zhu, 2005; Song *et al.*, 2007) and also to the Ni^II^ complex of 2,4-dihydroxybenzoate (Yang *et al.*, 2006).

Each dinuclear unit are connected through O-H···O hydrogen bonds involving two water molecules resulting in a R^2^_4_(8) graph set motif ( Etter *et al.*, 1990, Bernstein *et al.*, 1995) and building up an infinite chain parallel to the (1 0 -1) plane. C-H···O interactions further stabilize the chain. (Table 1; Fig. 2).

### Experimental

An ethanol-water solution (20 ml, 1:3) containing 2-hydroxy-4-chlorobenzoic acid (0.173 g, 1 mmol), Na_2_CO_3_ (0.053 g, 0.5 mmol) and CuCl_2_.2H_2_O (0.085 g, 0.5 mmol) was refluxed for 6 h, then phenanthroline hydrate (0.99 g, 1 mmol) was added into the solution and the mixture was refluxed for further 0.5 h. After cooling to room temperature the solution was filtered. Single crystals of the title compound were obtained from the filtrate after one week.

### Refinement

Water H atoms were located in a difference Fourier map and refined as riding in as-found relative positions with U_iso_(H) = 1.5U_eq_(O). Other H atoms were placed in calculated positions with C—H = 0.93 Å and refined in riding mode with U_iso_(H) = 1.2U_eq_(C).

### Crystal data

**Table:**

[Cu_2_(C_7_H_3_ClO_3_)_2_(C_12_H_8_N_2_)_2_]·2H_2_O	*F*(000) = 876
*M**_r_* = 864.60	*D*_x_ = 1.629 Mg m^−^^3^
Monoclinic, *P*2_1_/*c*	Mo *K*α radiation, λ = 0.71073 Å
Hall symbol: -P 2ybc	Cell parameters from 5352 reflections
*a* = 8.1941 (17) Å	θ = 2.2–24.2°
*b* = 18.851 (4) Å	µ = 1.42 mm^−^^1^
*c* = 11.873 (3) Å	*T* = 294 K
β = 105.993 (8)°	Prism, blue
*V* = 1763.0 (6) Å^3^	0.33 × 0.30 × 0.22 mm
*Z* = 2	

### Data collection

**Table:**

Rigaku R-AXIS RAPID IP diffractometer	3163 independent reflections
Radiation source: fine-focus sealed tube	2162 reflections with *I* > 2σ(*I*)
graphite	*R*_int_ = 0.047
Detector resolution: 10.0 pixels mm^-1^	θ_max_ = 25.2°, θ_min_ = 2.1°
ω scans	*h* = −9→9
Absorption correction: multi-scan (*ABSCOR*; Higashi, 1995)	*k* = −22→22
*T*_min_ = 0.656, *T*_max_ = 0.730	*l* = −13→14
18894 measured reflections	

### Refinement

**Table:**

Refinement on *F*^2^	Primary atom site location: structure-invariant direct methods
Least-squares matrix: full	Secondary atom site location: difference Fourier map
*R*[*F*^2^ > 2σ(*F*^2^)] = 0.037	Hydrogen site location: inferred from neighbouring sites
*wR*(*F*^2^) = 0.103	H-atom parameters constrained
*S* = 1.03	*w* = 1/[σ^2^(*F*_o_^2^) + (0.0509*P*)^2^ + 0.5996*P*] where *P* = (*F*_o_^2^ + 2*F*_c_^2^)/3
3163 reflections	(Δ/σ)_max_ = 0.001
244 parameters	Δρ_max_ = 0.58 e Å^−^^3^
0 restraints	Δρ_min_ = −0.35 e Å^−^^3^

### Special details

**Table:**

Geometry. All esds (except the esd in the dihedral angle between two l.s. planes) are estimated using the full covariance matrix. The cell esds are taken into account individually in the estimation of esds in distances, angles and torsion angles; correlations between esds in cell parameters are only used when they are defined by crystal symmetry. An approximate (isotropic) treatment of cell esds is used for estimating esds involving l.s. planes.
Refinement. Refinement of *F*^2^ against ALL reflections. The weighted *R*-factor wR and goodness of fit *S* are based on *F*^2^, conventional *R*-factors *R* are based on *F*, with *F* set to zero for negative *F*^2^. The threshold expression of *F*^2^ > σ(*F*^2^) is used only for calculating *R*-factors(gt) etc. and is not relevant to the choice of reflections for refinement. *R*-factors based on *F*^2^ are statistically about twice as large as those based on *F*, and *R*- factors based on ALL data will be even larger.

### Fractional atomic coordinates and isotropic or equivalent isotropic displacement parameters (Å^2^)

**Table:**

	*x*	*y*	*z*	*U*_iso_*/*U*_eq_	
Cu	0.28977 (4)	0.501289 (19)	0.46012 (3)	0.04370 (15)	
Cl	0.68680 (17)	0.80306 (6)	0.36270 (13)	0.1107 (5)	
N1	0.2931 (3)	0.50654 (13)	0.6296 (2)	0.0456 (6)	
N2	0.1240 (3)	0.42264 (13)	0.4601 (2)	0.0480 (6)	
O1	0.2426 (3)	0.49404 (12)	0.2962 (2)	0.0596 (6)	
O2	0.2234 (3)	0.52593 (16)	0.1153 (2)	0.0750 (7)	
O3	0.4451 (3)	0.57734 (11)	0.47282 (18)	0.0507 (5)	
C1	0.2743 (4)	0.5384 (2)	0.2219 (3)	0.0555 (9)	
C2	0.3717 (4)	0.60392 (18)	0.2646 (3)	0.0512 (8)	
C3	0.4487 (4)	0.61966 (16)	0.3838 (3)	0.0472 (7)	
C4	0.5430 (4)	0.68293 (17)	0.4110 (3)	0.0577 (9)	
H4	0.5950	0.6941	0.4889	0.069*	
C5	0.5593 (5)	0.72807 (19)	0.3250 (4)	0.0718 (11)	
C6	0.4837 (5)	0.7141 (2)	0.2088 (4)	0.0851 (14)	
H6	0.4947	0.7455	0.1509	0.102*	
C7	0.3919 (5)	0.6528 (2)	0.1802 (3)	0.0711 (11)	
H7	0.3408	0.6431	0.1015	0.085*	
C8	0.3797 (4)	0.55026 (19)	0.7127 (3)	0.0569 (9)	
H8	0.4424	0.5868	0.6923	0.068*	
C9	0.3792 (5)	0.5429 (2)	0.8297 (3)	0.0728 (11)	
H9	0.4401	0.5745	0.8857	0.087*	
C10	0.2897 (5)	0.4896 (2)	0.8616 (4)	0.0765 (13)	
H10	0.2892	0.4847	0.9394	0.092*	
C11	0.1979 (5)	0.4418 (2)	0.7767 (3)	0.0635 (10)	
C12	0.1001 (6)	0.3835 (3)	0.7997 (4)	0.0854 (14)	
H12	0.0971	0.3746	0.8761	0.102*	
C13	0.0125 (6)	0.3412 (3)	0.7126 (5)	0.0876 (14)	
H13	−0.0510	0.3041	0.7306	0.105*	
C14	0.0135 (5)	0.3514 (2)	0.5930 (4)	0.0675 (11)	
C15	−0.0751 (6)	0.3107 (2)	0.4968 (5)	0.0882 (14)	
H15	−0.1426	0.2731	0.5076	0.106*	
C16	−0.0626 (5)	0.3259 (2)	0.3884 (5)	0.0849 (13)	
H16	−0.1223	0.2990	0.3247	0.102*	
C17	0.0394 (5)	0.38181 (19)	0.3716 (3)	0.0639 (10)	
H17	0.0486	0.3909	0.2966	0.077*	
C18	0.1106 (4)	0.40810 (17)	0.5688 (3)	0.0505 (8)	
C19	0.2019 (4)	0.45334 (17)	0.6605 (3)	0.0499 (8)	
O1W	0.0002 (4)	0.59767 (16)	−0.0734 (2)	0.0934 (9)	
H1A	0.0653	0.5735	−0.0124	0.140*	
H1B	−0.0941	0.5733	−0.0960	0.140*	

### Atomic displacement parameters (Å^2^)

**Table:**

	*U*^11^	*U*^22^	*U*^33^	*U*^12^	*U*^13^	*U*^23^
Cu	0.0459 (2)	0.0543 (2)	0.0309 (2)	−0.00153 (17)	0.01078 (16)	0.00139 (17)
Cl	0.1106 (10)	0.0650 (6)	0.1553 (13)	−0.0153 (6)	0.0347 (9)	0.0283 (7)
N1	0.0459 (14)	0.0589 (15)	0.0335 (14)	0.0089 (13)	0.0134 (11)	−0.0016 (13)
N2	0.0424 (15)	0.0544 (15)	0.0472 (17)	0.0028 (12)	0.0124 (12)	−0.0025 (13)
O1	0.0688 (16)	0.0716 (15)	0.0349 (13)	−0.0104 (12)	0.0085 (11)	−0.0014 (11)
O2	0.0772 (18)	0.117 (2)	0.0277 (14)	0.0076 (16)	0.0085 (12)	0.0011 (13)
O3	0.0577 (13)	0.0586 (13)	0.0357 (12)	−0.0060 (10)	0.0125 (10)	0.0031 (10)
C1	0.0470 (19)	0.081 (2)	0.038 (2)	0.0107 (18)	0.0103 (15)	0.0060 (18)
C2	0.0443 (18)	0.069 (2)	0.0419 (19)	0.0106 (16)	0.0144 (15)	0.0163 (16)
C3	0.0443 (18)	0.0539 (18)	0.046 (2)	0.0079 (15)	0.0176 (15)	0.0062 (16)
C4	0.057 (2)	0.0537 (19)	0.064 (2)	0.0052 (16)	0.0197 (18)	0.0051 (17)
C5	0.060 (2)	0.058 (2)	0.099 (3)	0.0067 (18)	0.025 (2)	0.025 (2)
C6	0.075 (3)	0.095 (3)	0.089 (3)	0.008 (2)	0.028 (3)	0.053 (3)
C7	0.063 (2)	0.099 (3)	0.052 (2)	0.010 (2)	0.0164 (19)	0.028 (2)
C8	0.056 (2)	0.071 (2)	0.042 (2)	0.0117 (17)	0.0109 (16)	−0.0050 (17)
C9	0.074 (3)	0.102 (3)	0.038 (2)	0.021 (2)	0.0075 (19)	−0.011 (2)
C10	0.078 (3)	0.119 (4)	0.040 (2)	0.034 (3)	0.028 (2)	0.013 (2)
C11	0.058 (2)	0.089 (3)	0.052 (2)	0.024 (2)	0.0296 (18)	0.018 (2)
C12	0.076 (3)	0.118 (4)	0.078 (3)	0.031 (3)	0.050 (3)	0.045 (3)
C13	0.074 (3)	0.089 (3)	0.117 (4)	0.009 (2)	0.055 (3)	0.040 (3)
C14	0.054 (2)	0.064 (2)	0.094 (3)	0.0047 (18)	0.035 (2)	0.016 (2)
C15	0.077 (3)	0.062 (2)	0.129 (5)	−0.013 (2)	0.034 (3)	0.004 (3)
C16	0.069 (3)	0.069 (3)	0.113 (4)	−0.014 (2)	0.019 (3)	−0.024 (3)
C17	0.057 (2)	0.062 (2)	0.069 (3)	0.0008 (18)	0.0128 (19)	−0.0129 (19)
C18	0.0417 (18)	0.0575 (19)	0.055 (2)	0.0093 (15)	0.0183 (16)	0.0081 (17)
C19	0.0462 (19)	0.062 (2)	0.047 (2)	0.0175 (16)	0.0226 (15)	0.0131 (16)
O1W	0.097 (2)	0.111 (2)	0.0617 (18)	0.0296 (18)	0.0041 (15)	0.0005 (16)

### Geometric parameters (Å, °)

**Table:**

Cu—O1	1.882 (2)	C8—C9	1.397 (5)
Cu—O3	1.895 (2)	C8—H8	0.9300
Cu—O3^i^	2.569 (2)	C9—C10	1.358 (6)
Cu—N1	2.007 (3)	C9—H9	0.9300
Cu—N2	2.011 (3)	C10—C11	1.405 (6)
Cl—C5	1.741 (4)	C10—H10	0.9300
N1—C8	1.331 (4)	C11—C19	1.406 (4)
N1—C19	1.360 (4)	C11—C12	1.430 (6)
N2—C17	1.332 (4)	C12—C13	1.346 (6)
N2—C18	1.353 (4)	C12—H12	0.9300
O1—C1	1.292 (4)	C13—C14	1.435 (6)
O2—C1	1.241 (4)	C13—H13	0.9300
O3—C3	1.331 (4)	C14—C15	1.401 (6)
C1—C2	1.482 (5)	C14—C18	1.409 (5)
C2—C7	1.404 (4)	C15—C16	1.350 (6)
C2—C3	1.413 (4)	C15—H15	0.9300
C3—C4	1.409 (5)	C16—C17	1.393 (5)
C4—C5	1.363 (5)	C16—H16	0.9300
C4—H4	0.9300	C17—H17	0.9300
C5—C6	1.374 (6)	C18—C19	1.423 (5)
C6—C7	1.370 (6)	O1W—H1A	0.8969
C6—H6	0.9300	O1W—H1B	0.8747
C7—H7	0.9300		
			
O1—Cu—O3	94.54 (9)	C2—C7—H7	118.6
O1—Cu—N1	169.27 (10)	N1—C8—C9	121.9 (4)
O3—Cu—N1	93.42 (10)	N1—C8—H8	119.1
O1—Cu—N2	90.01 (10)	C9—C8—H8	119.1
O3—Cu—N2	175.25 (9)	C10—C9—C8	120.1 (4)
N1—Cu—N2	81.90 (11)	C10—C9—H9	120.0
O1—Cu—O3^i^	101.17 (9)	C8—C9—H9	120.0
O3—Cu—O3^i^	85.40 (9)	C9—C10—C11	119.8 (4)
N1—Cu—O3^i^	86.62 (8)	C9—C10—H10	120.1
N2—Cu—O3^i^	95.07 (9)	C11—C10—H10	120.1
C8—N1—C19	118.5 (3)	C10—C11—C19	116.9 (4)
C8—N1—Cu	129.1 (2)	C10—C11—C12	125.0 (4)
C19—N1—Cu	112.2 (2)	C19—C11—C12	118.1 (4)
C17—N2—C18	118.3 (3)	C13—C12—C11	121.2 (4)
C17—N2—Cu	129.1 (3)	C13—C12—H12	119.4
C18—N2—Cu	112.4 (2)	C11—C12—H12	119.4
C1—O1—Cu	129.5 (2)	C12—C13—C14	122.2 (4)
C3—O3—Cu	123.5 (2)	C12—C13—H13	118.9
O2—C1—O1	119.9 (3)	C14—C13—H13	118.9
O2—C1—C2	120.3 (3)	C15—C14—C18	116.3 (4)
O1—C1—C2	119.8 (3)	C15—C14—C13	126.2 (4)
C7—C2—C3	118.0 (3)	C18—C14—C13	117.5 (4)
C7—C2—C1	117.4 (3)	C16—C15—C14	120.2 (4)
C3—C2—C1	124.6 (3)	C16—C15—H15	119.9
O3—C3—C4	117.2 (3)	C14—C15—H15	119.9
O3—C3—C2	124.6 (3)	C15—C16—C17	120.3 (4)
C4—C3—C2	118.2 (3)	C15—C16—H16	119.9
C5—C4—C3	121.2 (4)	C17—C16—H16	119.9
C5—C4—H4	119.4	N2—C17—C16	121.8 (4)
C3—C4—H4	119.4	N2—C17—H17	119.1
C6—C5—C4	121.5 (4)	C16—C17—H17	119.1
C6—C5—Cl	119.1 (3)	N2—C18—C14	123.2 (3)
C4—C5—Cl	119.4 (3)	N2—C18—C19	116.4 (3)
C5—C6—C7	118.5 (3)	C14—C18—C19	120.4 (3)
C5—C6—H6	120.8	N1—C19—C11	122.8 (3)
C7—C6—H6	120.8	N1—C19—C18	116.6 (3)
C6—C7—C2	122.7 (4)	C11—C19—C18	120.6 (3)
C6—C7—H7	118.6	H1A—O1W—H1B	105.1

Symmetry codes: (i) −*x*+1, −*y*+1, −*z*+1.

### Hydrogen-bond geometry (Å, °)

**Table:**

*D*—H···*A*	*D*—H	H···*A*	*D*···*A*	*D*—H···*A*
O1W—H1A···O2	0.90	1.92	2.817 (4)	175
O1W—H1B···O2^ii^	0.88	2.13	2.921 (4)	150
C10—H10···O2^iii^	0.93	2.42	3.277 (5)	153
C17—H17···O1W^ii^	0.93	2.58	3.487 (4)	166

Symmetry codes: (ii) −*x*, −*y*+1, −*z*; (iii) *x*, *y*, *z*+1.
